# An evaluation of clinical, radiological and three-dimensional dental tomography findings in ectodermal dysplasia cases

**DOI:** 10.4317/medoral.20303

**Published:** 2015-02-07

**Authors:** Mehmet-Sinan Doğan, Michele Callea, İzzet Yavuz, Orhan Aksoy, Gabriella Clarich, Ayşe Günay, Ahmet Günay, Sedat Güven, Michele Maglione, Zeki Akkuş

**Affiliations:** 1 Department of Pediatric Dentistry, Faculty of Dentistry, Dicle University, Diyarbakir, Turkey; 2 Institute for Maternal and Child Health - IRCCS “Burlo Garofolo”- Trieste, Italy; 3 Department of Orthodontics, Faculty of Dentistry, Dicle University, Diyarbakir, Turkey; 4 Department of Periodontology, Faculty of Dentistry, Dicle University, Diyarbakir, Turkey; 5 Department of Prosthodontics, Faculty of Dentistry, Dicle University, Diyarbakir, Turkey; 6 University of Trieste, Department of Medical, Surgical and Health Sciences, Trieste, Italy; 7 Department of Biostatistics, Faculty of Medicine, Dicle University, Diyarbakir, Turkey

## Abstract

**Background:**

This study aimed to review the results related to head and jaw disorders in cases of ectodermal dysplasia. The evaluation of ectodermal dysplasia cases was made by clincal examination and examination of the jaw and facial areas radiologically and on cone-beam 3-dimensional dental tomography (CBCT) images.

**Material and Methods:**

In the 36 cases evaluated in the study, typical clinical findings of pure hypohidrotic ectodermal displasia (HED) were seen, such as missing teeth, dry skin, hair and nail disorders. CBCT images were obtained from 12 of the 36 cases, aged 1.5- 45 years, and orthodontic analyses were made on these images.

**Results:**

The clinical and radiological evaluations determined, hypodontia or oligodontia, breathing problems, sweating problems, a history of fever, sparse hair, saddle nose, skin peeling, hypopigmentation, hyperpigmentation, finger and nail deformities, conical teeth anomalies, abnormal tooth root formation, tooth resorption in the root, gingivitis, history of epilepsy, absent lachrymal canals and vision problems in the cases which included to the study.

**Conclusions:**

Ectodermal dysplasia cases have a particular place in dentistry and require a professional, multi-disciplinary approach in respect of the chewing function, orthognathic problems, growth, oral and dental health. It has been understood that with data obtained from modern technologies such as three-dimensional dental tomography and the treatments applied, the quality of life of these cases can be improved.

**Key words:** Ectodermal dysplasia, three-dimensional dental tomography.

## Introduction

Ectodermal Dysplasias (EDs) are a group of inherited disorders affecting ectodermal derived tissues (hair, nails, teeth, skin and sweat glands). Hypohidrotic ectodermal dysplasia (HED) represents one of the major types of EDs and is due to mutations in the EDA (MIM *300451), EDAR (MIM *604095) and EDARADD (MIM *606603) genes. The inheritance can be X-linked (XLHED; MIM#305100) or autosomal either dominant (MIM#129490) or recessive (MIM#305100) (1-4). Ectodermal dysplasia (ED) shows X-related recessive, autosomal dominant and autosomal recessive transfer (pattern of inheritance). ED is a complex group of genetic disorders, which develops from the ectoderm layer with two or more tissue anomalies resulting in heterogeneous characteristics (1-3).

In most cases, tissues originating from the ectoderm are affected (3). Incidence has been reported as 1 in 100,000 births of all races.

Typical findings of ED are seen as congenital malformations in the teeth, hair, nails and sweat glands. Reduced vertical facial height and depth, flattened nose root, prominent forehead and full lips are seen. Findings inside the mouth can be seen as thin alveolar ridges and anodontia or hypodontia of the primary and permanent teeth (1-7). Thus, because of the lack of teeth and facial and dental appearance, these cases may experience social and psycho social problems (7). The syndrome type may often be accompanied by deafness, skeletal anomalies, mental retardation, ichthyosis, palmoplantar keratoderma, eye abnormalities, facial deformities, cleft of the palate and the lip and other systemic findings (7). Almost 200 different ED types have been defined (3,4).

Ectodermal dysplasia is often defined in three major groups of anhydrotic (Christ-Siemens-Touraine syndrome), hypohydrotic and hydrotic (Clouston syndrome). Anhydrotic ectodermal dysplasia is characterized by autosomal recessive transfer and absence of sweat and fat glands and is more often seen than the other types. These findings are partially seen in a more mild form in the hypohydrotic type. In the hydrotic type, which is transferred as autosomal dominant, the sweat and fat glands are of normal formation (1,3,4,6).

Freire-Maia ectodermal dysplasia is categorised in 2 main groups (8,9).

In Group A; defects are found in at least two of the structures originating from the ectoderm, such as hair, nails, teeth and sweat glands. The A group is subdivided into 11 subgroups, according to the involved structures: 1-2-3-4 (hair-teeth-nails-sweat glands); 1-2-3 (hair-teeth-nails); 1-2-4 (hair-teeth-sweat glands); 1-3-4 (hair-nails-sweat glands); 2-3-4 (teeth-nails-sweat glands); 1-2 (hair-teeth); 1-3 (hair-nails); 1-4 (hair-sweat glands); 2-3 (teeth nails); 2-4 (teeth-sweat glands); 3-4 (nails-sweat glands).

In Group B; defects are found in only one of the structures (hair, teeth, nails, and sweat glands) plus another ectodermal defect.

Although there is normal dimensional growth in the basal plane of the jaw bones of cases, the alveolar crests are thin because of missing teeth and the crests cannot develop sufficiently vertically. That alveolar crests cannot develop when there are missing teeth reduces the vertical size and causes a convex appearance of the lips. In the upper jaw, the palate is generally deep and cleft palate or lips may be seen. As complete absence of salivary glands is very rarely seen, dry mouth is not seen in all cases (5,10). However, in cases with reduced saliva secretion, periodontal problems have also been observed. The content of saliva in the mouth cavity forms an important defense mechanism, so if saliva is reduced, bacterial plaque and food remnants easily accumulate. In addition, the oxygen found in saliva prevents the proliferation of anaerobic bacteria which are agents of periodontal diseases and protect against halitosis. The absence of all these factors makes an individual vulnerable to periodontal diseases (11,12).

Early multidisciplinary dental intervention is required in terms of the conservation and development of the chewing function and optimal facial appearance. The aim of prepared prostheses is to protect existing teeth as well as preventing resorption which may occur in the alveolar crests (5).

Early intervention in children gives the opportunity for normal development of chewing and swallowing, normal formation of the normal temperomandibular joint function and general healthy growth and development (10).

## Material and Method

This study was conducted retrospectively by examination of the information obtained from pure hypohidrotic ectodermal displasia (HED) cases who presented at our clinic between 2006 and 2013. A total of 36 cases (19 female, 17 male) aged 1.5-45 years were examined for evaluation. In the clinical and radiological evaluations, hair, nails, skin, nose, sweat glands and similar malformations were examined. The patients and their guardians were informed of signs related to HED.

We have Informed Consent that its mean all of patient have information about taking CBCT and use of them for treatment plan and scientific aim.

“Study has been approved by an ethics committee” mean our research approved by local ethics committee.

**Figure 1:**
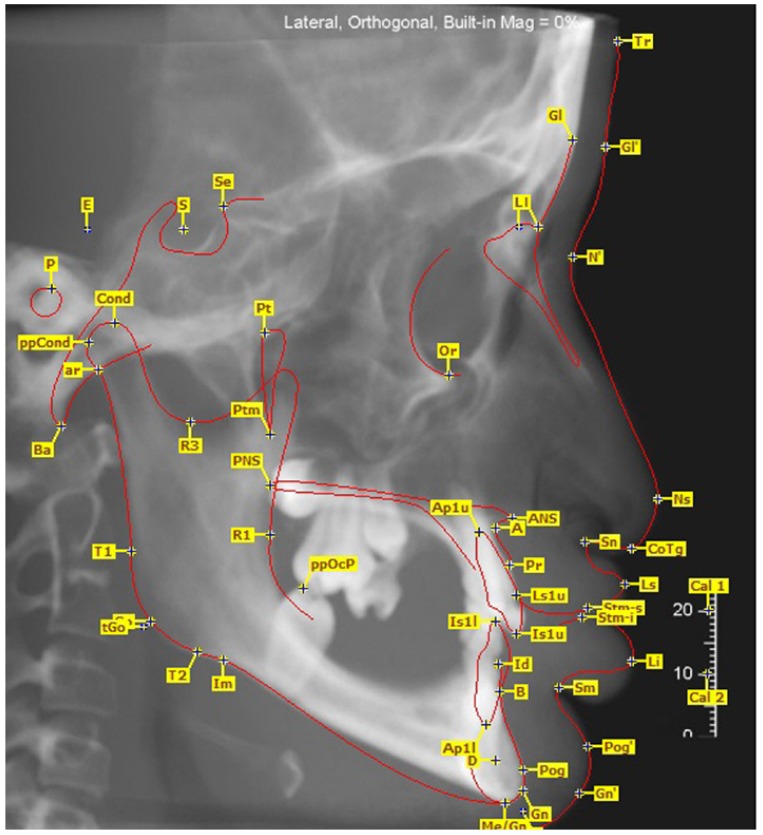
Lateral cephalometric image obtained with CBCT and the reference points.

**Figure 2:**
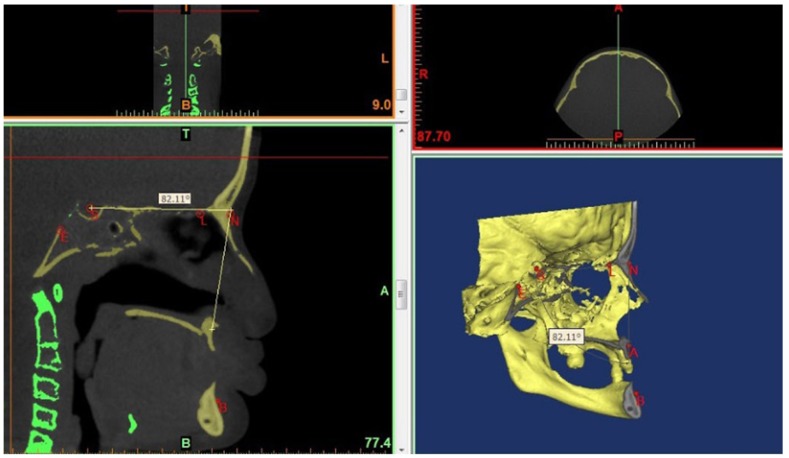
a,b. Saggital slice image for cephalometric analysiswith the Mimics program (a), 3-D image obtained with CBCT (b).

**Figure 3:**
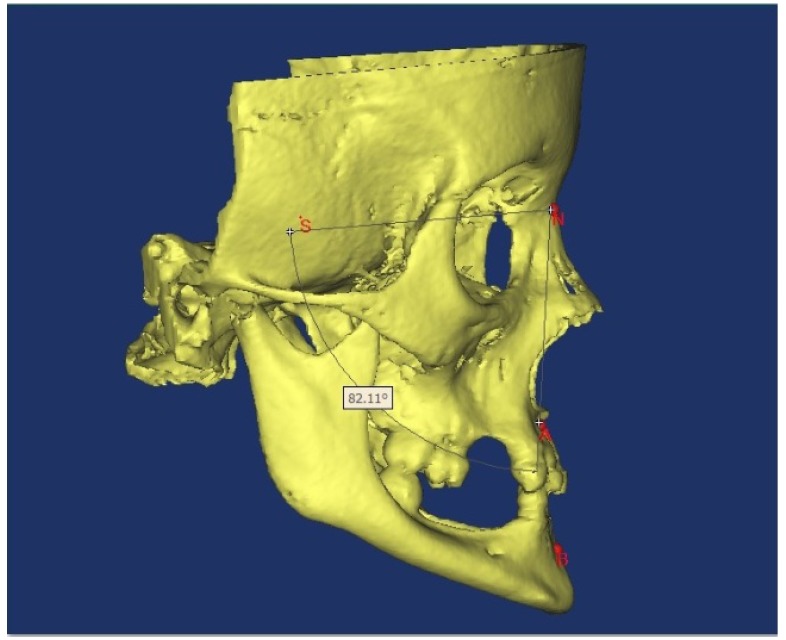
3-D image obtained with CBCT for cephalometric analysis.

Angles showing vertical development and angles showing sagittal development were measured and soft tissue was also evaluated with the Björk cephalometric Sassouni Sapmermans, Tweed, Steiner, McNamara, Subtelyn and Ricketts analyses. The obtained values were statistically compared with normal values using Student’s t-test. Of the total cases included in the study, cone beam computerized tomography (CBCT) images were obtained from 12 (Figs. 1,2,3).

The CBCT device (i-CAT®, Model 17-19, Imaging Sciences International, Hatfield, Pa USA) consists of a standard high-frequency fixed anode X-ray tube (120 kVp, 3-7 mA) and 19.2 x 23.8 cm amorphous silicone (a-Si) flat panel image detector, and uses a cone-shaped x-ray collimator with a 15-degree aperture centered on an x-ray area detector. It acquires raw data with a single 360° rotation in 8.9 seconds around the patient’s head, with a projection at every 1° step, captured by an amorphous silicone flat panel image detector and stored on a hard drive. After the volume obtained the reconstruction time was ~ 30 seconds. All images were obtained with 440 projections and the voxel dimension in the reconstructed image is 0.3 X 0.3 X 0.3 mm and reconstruction shape was cylindrical.

## Results

The clinical and radiological evaluations determined hypodontia or oligodontia in all the cases included in the study. HED findings were found in the siblings of 17 cases, 19 cases had breathing problems, 31 cases had sweating problems, 26 cases had a history of fever, 34 cases had sparse hair, 24 cases had saddle nose, 30 cases had skin peeling, 5 cases had hypo pigmentation, 15 cases had hyper pigmentation, 29 cases had finger and nail deformities, 25 cases had conical teeth anomalies, 3 cases had abnormal tooth root formation, 1 case had tooth resorption in the root, 21 cases had gingivitis, 1 case had a history of epilepsy, 2 cases had absent lachrymal canals and 1 case had vision problems ( Table 1, Table 2).

**Table 1:**
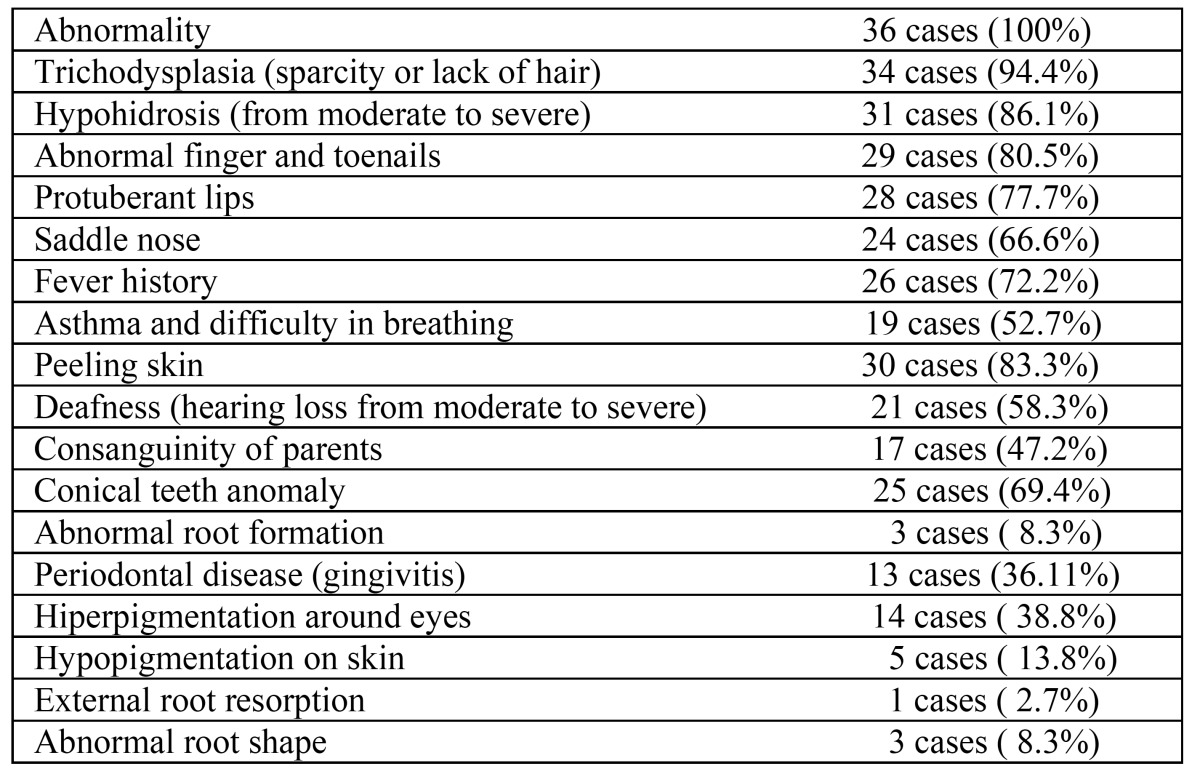
Clinical Findings of the 36 Study Cases.

**Table 2:**
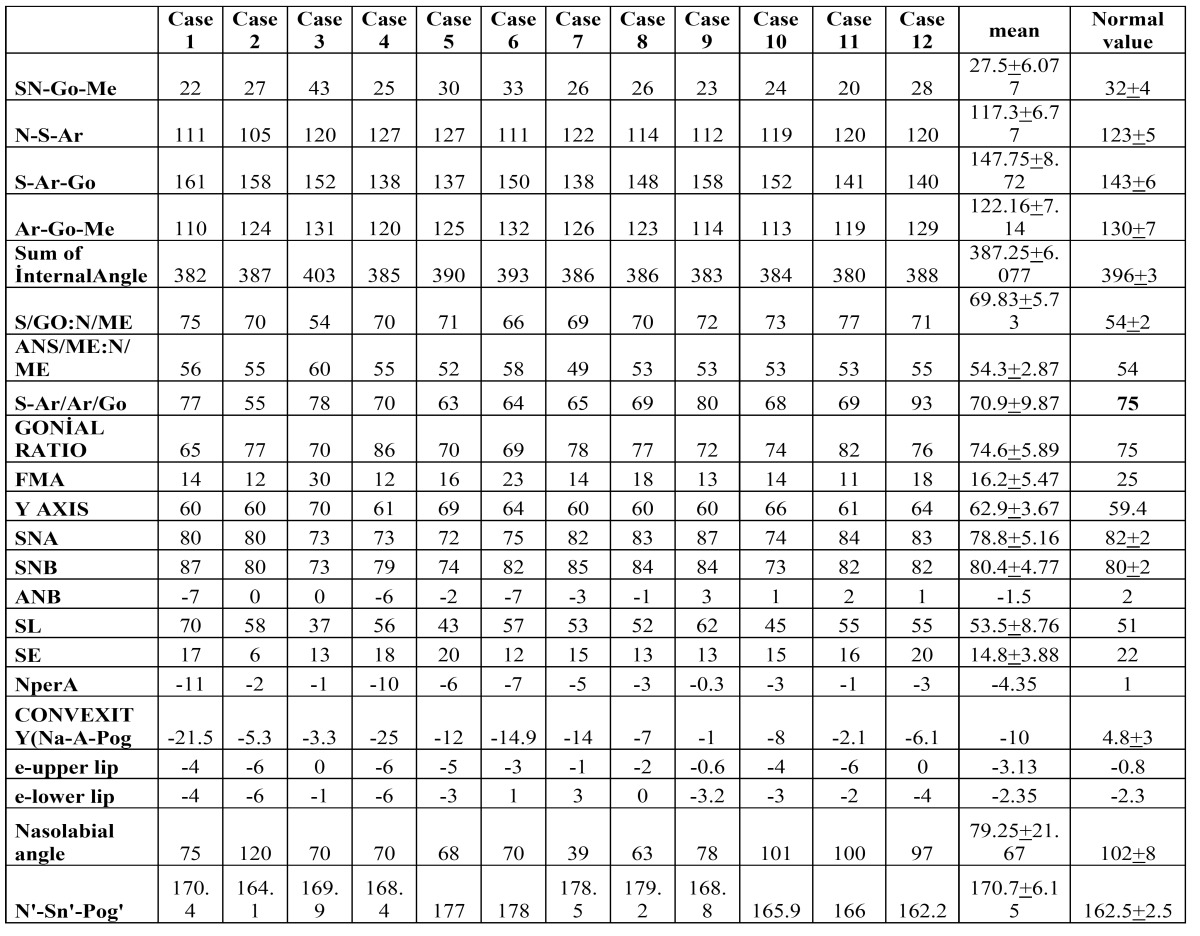
Values obtained from the measurements of the Björk cephalometric Sassouni Sapmermans, Tweed, Steiner, McNamara, Subtelyn and Ricketts analyses of 12 cases.

On the CBCT images obtained from 12 of the total 36 cases, Björk cephalometric Sassouni Sapmermans, Tweed, Steiner, McNamara, Subtelyn and Ricketts analyses were made. The analysis results were compared with normal values.

## Discussion

In ectodermal dysplasia, more than two tissues are affected together with the skin and anomalies are formed in the hair, teeth, nails and sweat glands (4,7). In a study by Dellavia *et al*, 62% of cases were determined to have conical central teeth (13). In the current study, the rate of conical tooth anomaly was 69.4%.

Impairments in the appearance of head and facial structures are also seen. It has been reported that there may be disorders such as allergic rhinitis and sinusitis (4). In the current study, hair, tooth and nail anomalies were seen in all the cases.

Ear debris related to epithelium migraton and an excessive amount of keratin debris often recurs. To prevent chronic otitis and hearing problems, in some cases it has been reported to be necessary to make a small incision in the eardrum with a route to the middle ear to empty collected fluid in the middle ear or to irrigate the middle ear (eardrum parasynthesis, tympanotomy, myringotomy). In recent studies, recurrent otitis media has been reported in 49% of cases and hearing problems in 43%. These cases have been reported to be predisposed to acute pharyngolaryngitis and pulmonary infections (14). In the current study, 58.3% of the cases were determined to have ear-related problems.

In an epidemiological study by M. Nguyen-Nielsen *et al* of 1224 cases of ectodermal dysplasia aged 11-18 years, 79.4% were found to have teeth anomalies, 11.4% hypotrichosis and 5.9% hypohidrosis (15). Yavuz *et al* determined absent teeth in all 15 cases of a study, trichodysplasia in 12 cases, hypohidrosis in 13 cases and abnormal fingers and nails in 12 cases (3). In the current study, teeth anomalies were determined in all cases. Hypohydrosis was seen in 86.1%, trichodysplasia in 94.4% and nail and finger anomalies in 80.5%.

In ectodermal dysplasia cases, the most absence of teeth has been reported to be in the mandible and in males. It has also been reported that in all affected males there may be abnormal crown formation of the maxillary incisor teeth, abnormal root formation of the molar teeth and taurodontism may be seen in the majority. Generally in heterozygote females, teeth are observed to be significantly smaller than normal (16,17).

Reduced saliva secretion and loss of alveolar bone increase the predisposition of ectodermal dysplasia patients to periodontal diseases (11,12,18). Gingivitis was determined in the current study patient group at 36.11%.

Lexner *et al* conducted a study on teeth anomalies in ectodermal dysplasia males and heterozygote females using orthopantomographic radiographs and dental casts. Widespread anomalies were determined in crown morphology in both males and females (16). In an epidemiological study by Ruhin *et al*, 2 cases were determined with anadontia, 5 cases were determined with fewer than 10 teeth and 9 cases with more than 10 teeth. In cephalometric analyses, cases with serious maxillary hypotrophy and absent teeth were reported as Class 3 (19). In the current study, 11 cases were found to have fewer than 10 teeth and 3 cases had abnormal root formation. In a clinical study by Ruhin *et al* of ectodermal dysplasia cases, severe hypodontia was determined. It was also reported that all the cases in the study had absent teeth, skin vesicles, thinning hair and nail anomalies. Mandibular protusion, maxillary retrusion and facial concavity together with a short facial appearance were also reported in most cases (19). In the current study, hair and nail anomalies were determined in 29 cases. In a cephalometric study, maxillary hypo trophy and retrusion, anterior mandibular advance and reduced facial height were determined to have normalised with a prosthesis (19). In most cases, severe lack of teeth, reduced alveolar crest, anterior and posterior reduced facial height, maxillary hypo trophy and mandibular protusion have been determined.

In an anthropometric analysis, concavity and low facial height were seen in 8 cases (19). In the current study, saddle nose was determined in 24 cases and protuberant lips in 28 cases. The total SN-Go-Me, N-S-Ar, Ar-Go-Me olcak internal angles found as a result of the analyses made on the CBCT images obtained from 12 cases in the current study, were compared as SGO/NMe, FMA, Y axis, SE, nasolabial and soft tissue convexity angle values with normal values and a statistically significant difference was determined (Student’s t-test, *p*<0.05). When the total SN-Go-Me and internal angles were evaluated, low vertical size was determined in the patients. According to the Steiner cephalometric analysis, ANB was found to be mean -1.5. This value showed a tendency of the cases to skeletal class 3. According to the McNamara analysis, Nper-A was found to be mean -4.35, which showed that the cases had a concave skeletal angle.

In a clinical study of 35 cases by Sforza *et al*, facial convexity in both the horizontal and sagittal planes of ED cases was found to be significantly greater than normal. Mandibular corpus convexity in the horizontal plane was found to be similar to this result. Analysis of the cases revealed that the naso-labial angle was significantly reduced. In addition, the z-score was found to be negative for both the right and left gonial angles, the inter labial angles and the nasal convexity (20). According to the Ricketts soft tissue analysis made on the obtained CBCT images, normal values were determined in the upper and lower lips. The naso-labial angle was reduced compared to the normal value (79.25, normal:102±8). When soft tissue convexity was evaluated (N’-Sn’-Pog’), the soft tissue convexity value was determined to be increased (170.7, normal: 160-165).

## Conclusions

Ectodermal dysplasia is a heterogeneous group of hereditary malformations and irregularities which have similar findings. However, within the classification of ectodermal dysplasia, most cases have not yet been genetically defined. In this study, it was determined that ectodermal dysplasia not only creates tissue malformations but that the quality of life of patients is also affected.

As the images obtained with cone-beam three-dimensional dental tomography are clear and the most close to reality, orthodontic measurements made on these patients with this method can be considered to be reliable and thus the diagnosis and treatment will be more successful.

Although cone-beam 3-D dental tomography is an innovative and promising technology, as the amount of effective radiation is higher than that of traditional radiographs, it may not be correct to claim that this should be the technique required in the first stages of diagnosis and treatment for cases of ectodermal dysplasia.

However, as cone-beam 3-D dental tomography is superior to traditional and digital imaging methods, it can be considered to allow the possibility of more accurate dental treatment and orthodontic analysis as images obtained from ectodermal dysplasia cases are close to reality.

As ectodermal dysplasia cases have a particular place in dentistry, with treatments made with a professional, multidisciplinary approach using modern technologies such as three-dimensional dental tomography, the quality of life of these cases can be improved.
